# Keeping in touch with contact inhibition of locomotion

**DOI:** 10.1016/j.tcb.2010.03.005

**Published:** 2010-06

**Authors:** Roberto Mayor, Carlos Carmona-Fontaine

**Affiliations:** Department of Cell and Developmental Biology, University College London, Gower Street, London WC1E 6BT, UK

## Abstract

Contact inhibition of locomotion (CIL) is the process by which cells *in vitro* change their direction of migration upon contact with another cell. Here, we revisit the concept that CIL plays a central role in the migration of single cells and in collective migration, during both health and disease. Importantly, malignant cells exhibit a diminished CIL behaviour which allows them to invade healthy tissues. Accumulating evidence indicates that CIL occurs *in vivo* and that regulation of small Rho GTPases is important in the collapse of cell protrusions upon cell contact, the first step of CIL. Finally, we propose possible cell surface proteins that could be involved in the initial contact that regulates Rho GTPases during CIL.

## Social behaviour of migratory cells

In multicellular organisms, cell migration is essential for normal development and is required throughout life for numerous processes, including wound healing and responses to infections. Disregulation in the control of cell migration can lead to, or exacerbate, human diseases such as cancer, atherosclerosis and chronic inflammatory pathologies. More than a century of research in this area has generated a detailed morphological description of moving cells and this has allowed researchers to deepen their understanding of the molecular mechanisms that control cell polarity and cell protrusions during migration. An important concept to emerge is the idea that most cells do not move as isolated entities *in vivo* but rather interact with their neighbours during migration. Even cells of the immune system that can migrate singly have to interact with other non-motile cells along their migratory paths. Thus, cells must have their locomotory machinery adapted to these constant interactions. This has prompted scientists for decades to try to investigate the ‘social behaviour of cells’ [1]. However, how cells interact during migration is still not fully understood.

More than five decades ago, Abercrombie and Heaysman found that the direction of migration of fibroblasts cultured *in vitro* was affected by their interaction with other cells [1]. They called this process ‘contact inhibition of locomotion’ (CIL, see Refs. [2,3], Box 1) and it was proposed as an explanation for wound healing of epithelia, as this inhibition of cell contact dependent cell migration was released during wound healing, allowing the migration of the cells at the border of the wound [3,4]. The potential importance of this idea became immediately apparent when they observed that malignant mesenchymal cells showed a reduced CIL response, being able to invade fibroblast cultures in what was compared to invasive metastasis (see Refs [3–5]). Nonetheless, several factors led to a gradual loss of interest in the basis of this phenomenon. The molecular mechanism that orchestrates CIL has remained elusive for decades with only few recent advances [6–9]. This is partly owing to the fact that evidence for CIL occurring *in vivo* has been sparse [9–11]. Moreover, a different process, involving cell division rather than locomotion, was also named contact inhibition, leading to some confusion in the literature (see Glossary). Finally, there are also some common misconceptions, for example that CIL only happens when cells collide, or that its sole function is to inhibit migration.

In this article, we revisit the data that indicate CIL is a crucial mechanism for cell migration *in vivo*. Recent advances support a view of CIL as a general mechanism of local inhibition of cell protrusions in migratory cells. This leads to directional migration via the redirection of colliding single cells. Also, it is possible that CIL leads to directional movement in collective cell migration via promotion of coherence among cells. Thus, we propose that CIL could play an important role in coordinating the migration of this recognized mode of migration in embryonic and cancer cells [12,13]. Ongoing improvements in live imaging allow us to analyze CIL *in vivo* to test its importance in this context. We will review the few molecules reported to be involved in CIL. Moreover, recent advances in our understanding of the molecular bases of cell migration will allow us to propose more candidate molecules that mediate CIL and a molecular link among them.

## What is CIL?

**Figure 1:**
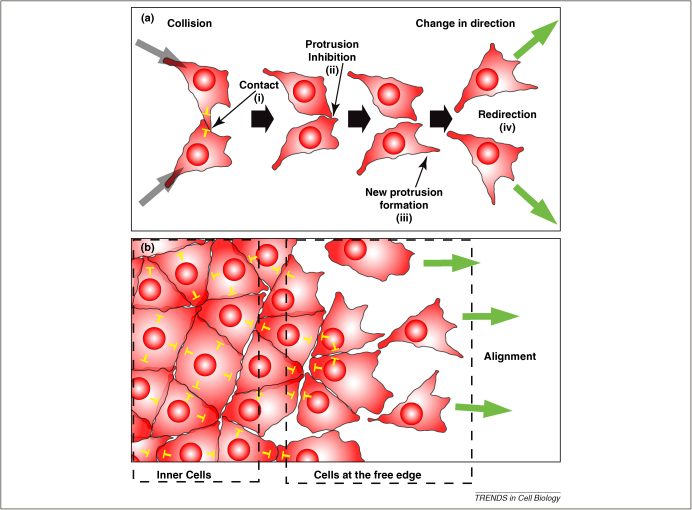
CIL in isolated cells **(a)** or in a group of cells **(b)**. CIL is represented by yellow inhibitory arrows. **(a)** Collision between single cells leads to collapse of cell protrusion and a change in the direction of migration (green arrows). The four steps of CIL are shown with roman numerals (see main text for details). **(b)** CIL in a group of cells. CIL between inner cells leads to inhibition of protrusions, whereas CIL between the leader cells, at the free edge, can lead to cell polarization of the leaders (green arrows) and directional migration.

**Figure 2:**
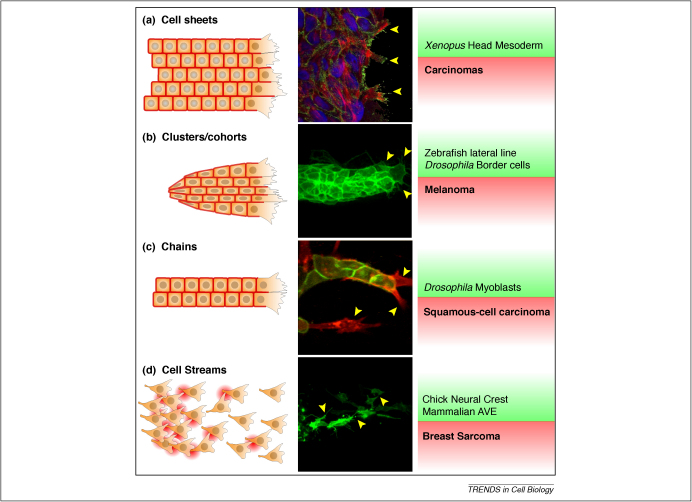
Examples of collective cell migration. First column: schematic representation of different migratory types. The regions where cells are interacting are depicted as a red border. Second column: examples. **(a)** Intestinal epithelial cells. From Ref. [94], used with permission. **(b)** Zebrafish lateral line. Reprinted from: Haas *et al.* (2006) Chemokine signaling mediates self-organizing tissue migration in the zebrafish lateral line, *Developmental Cell* 10, 673–680, with permission from Elsevier. **(c)** Fibroblast-leaded squamous cell carcinoma invasion. Adapted by permission from Macmillan Publishers Ltd: *Nature Cell Biology*[32]. **(d)** Avian neural crest. Reprinted from: Rupp *et al.* (2007) A role for RhoA in the two-phase migratory pattern of post-otic neural crest cells, *Developmental Biology* 311, 159–171, with permission from Elsevier. Yellow arrowheads show localised protrusion formation. Third column: examples of these different types of migration in health (green background) and disease (red background). AVE: anterior visceral endoderm.

The concept of CIL describes the observed behaviour of a cell to change the direction of its movement after contact with another cell (Box 1). The typical sequence of cell activities implicated in CIL are: (i) cell–cell contact, (ii) inhibition of cell protrusive activities at the site of contact, (iii) generation of a new protrusion away from the site of cell contact and (iv) migration in the direction of the new protrusion (Figure 1a). However, this sequence can be modified by different factors. For example, one of the cells might not be responsive to the other and thus, only one of the cells will be redirected. The number of surrounding cells can also alter the outcome of CIL. This four-step sequence is usually observed when individual cells, such as two fibroblasts, collide. However, in a sheet of cells only the cells at the free edge will produce lamellipodia whereas cells in contact with others at the centre of the cluster will generate smaller and more transient protrusions, if any. In this case, CIL will lead to the inhibition of cell protrusions of the inner cells in a cluster (Figure 1b). If a cluster of packed cells has a free edge, only the cells at the leading edge will produce protrusions. This can lead to directional migration of the whole cluster (Figure 1b), [2,4]. As a consequence of this behaviour, cells exhibiting CIL do not crawl over their neighbours leading to monolayer formation in groups and to scattering in single cells.

The lamellipodium has been described as the typical locomotory apparatus used to sense the adjacent cells during CIL; however, it is possible that other cell protrusions are also involved in this phenomenon (Box 2). For example, it has been recently proposed that filopodia could be the actual sensory structure in CIL [14]. This could mediate a type of CIL that would operate at longer distances than the cell body size and would probably involve collapse of protrusions but not necessarily a contact between the cell bodies. In fact, analysis of neural crest (NC) migration *in vivo* shows that these cells establish filopodia-like contacts with neighbouring cells and that this contact is sufficient to promote CIL [9,15].

### CIL can control cell polarity

Spatial cues such as chemoattractants are usually considered to explain the persistent orientation of cell polarity that leads to directional migration [16]. CIL can also contribute to cell polarization because the cells form their protrusions away from the cell–cell contact. Molecules localized to the cell–cell contact will be absent from the leading edge and therefore contribute to polarity [17–19]. Thus, CIL does not only halt migration of cells but also allow cells to re-polarize and migrate in a new direction, serving as another type of spatial cue [20]. This is crucial during the migration of cells that disperse from an original common location, which is a frequent feature found in embryo development. For example, endodermal cells from mouse and zebrafish embryos are initially localized in a specific region of the forming body from where they disperse to colonize their final destinations (respiratory and digestive tracts) [21,22], or the myeloid cells, formed in the ventral region of the *Xenopus* embryo that need to scatter along the entire epidermis [23]. When migration of all these kinds of cells is carefully analysed, a clear suggestion of CIL behaviour is observed (refer to the supplementary videos in the aforementioned references). Cell collisions lead to a change in the direction of migration, upon which cells move away from each other. In addition, these cells rarely overlap and when protrusions are visible, they seem to retract upon contact. These features suggest that these migratory cells, and probably many others, exhibit CIL *in vivo.* The observation that cells are constantly contacting each other argues against the role of a chemorepellent as a mechanism of dispersion; although it is probable that a combination of different mechanisms drives cell migration in this and other processes during development.

Several mechanisms are known to operate during cell migration *in vivo* such as random walk, chemoattraction and cell intercalation [21,24]. However, time lapse analysis of these migrating cells is consistent with CIL being an additional mechanism that contributes to cell migration. For example, the migration of Cajal-Retzius cells, a transient neuronal population crucial for the development of the brain cortex, is controlled by the apposed meningeal membranes, which produce and secrete the chemokine Cxcl12 [25]. However, this chemokine seems to be uniformly distributed along the migratory space and what provides the directionality in the dispersion of these cells are ‘contact-inhibitory interactions’ [25], which correspond to CIL. Indeed the assay used to characterize the interactions between these cells is the same as the one used by Abercrombie and Heaysman when CIL was initially described [2]. These observations suggest that CIL could be a general migratory mechanism that co-exists with processes such as chemoattraction, random-walk and cell intercalation to re-set the polarity of migratory cells.

### Collective cell migration: migratory ensembles require harmonic movements

In multicellular organisms, cells often move in groups rather than as singular cells. Cell migration in loosely or closely associated groups has been called collective cell migration (reviewed in Refs [12,13,26]). Collective cell migration is now a widely recognized mode of migration during embryogenesis and cancer. Both collective cell migrations and CIL are defined by the ability of cells to interact with their neighbours during migration and it is probable that these two processes are linked. There is a wide variety of collective cell migration, from sheets of migrating cells found in carcinomas and in head mesoderm of amphibian embryos (Figure 2a, [6,27,28]), to closely associated clusters of cells such as the migration of the lateral line in zebrafish, border cells in *Drosophila* embryos or melanomas (Figure 2b, [29,30]). Other cells are organized in chains such as *Drosophila* myoblasts or squamous cell carcinoma (Figure 2c, [31,32]). Another example of this is the migration of endothelial cells during sprouting in angiogenesis, in which inhibition of cell protrusion between the cells and presence of large lamellipodia and filopodia in the leader cells has been compared to CIL [33]. Another mode of collective cell migration has been called streaming (Figure 2d), and has been found in the migration of neural crest cells, mammalian endoderm and possibly in some breast carcinomas [9,15,34,35]. In this type of migration, the cells move as a loose cluster in which individual cells can be identified but are constantly interacting with each other. Interestingly, it has been shown that during neural crest migration, an example of cell streaming, cells make local and transient contacts which are required for CIL [9,15].

Despite the diversity of types of collective migration, there is a common theme for all of them: they all have major protrusions at the leading edge and show a high degree of organization and coordination during migration, which are features of CIL [6,9]. Although the degree of inhibition of cell protrusions between cells is variable and in some cases cryptic protrusions are observed between cells [36,37], it is tempting to speculate that the inhibition of protrusions in cell clusters during collective cell migration is based on CIL. If so, the two types of CIL – in single cell migration (Figure 1a) and in collective movements (Figure 1b) – would represent two aspects of the same process. It has been shown that CIL plays a role in collective migration of mesenchymal cells, such as neural crest [9,15], but there is no evidence that it could have a similar role in migration of more epithelial cells. Further knowledge of the molecular basis of CIL is required to compare it with the molecular mechanism that inhibits cell protrusions during collective cell migration.

## Predictions for the molecular bases of CIL

The molecular mechanisms that regulate CIL are still unknown, which is largely owing to the lack of investigation and debate on this topic. Despite the sparse data available, it is still possible to dissect CIL into two core cellular mechanisms requiring two different types of molecular machineries. First, cells need to sense the contact with other cells. This mechanism needs to be mediated by molecules located at the cell surface and to have a cognate ligand/receptor pair on the surface of the contacting cell. Moreover, molecules mediating the contact are also required to be able to transduce the signal from the juxtaposed cell into the responding cell. This response is the second mechanism. Upon contact, cells require a mechanism that regulates the withdrawal of protrusions at the contact region followed by the formation of a new protrusion elsewhere. Thus, the second mechanism is basically a repolarization mechanism. Importantly, molecules involved in these two mechanisms have been described as required for proper CIL. However, these two mechanisms have not been directly linked to each other. In this section we will review this evidence and propose a possible molecular link between these findings. Also we propose other surface molecules that could be mediating CIL (Box 3).

### Cell surface molecules potentially involved in CIL

Different pieces of information suggest that molecules, usually linked with cell–cell adhesion, are likely to mediate CIL. Although at a first sight there might be an apparent contradiction between CIL and adhesion, there is actually a long-standing link between these two mechanisms [4]. Although CIL implies cell repulsion and dispersion, it also requires adhesion to strengthen the contact and to allow cell–cell signalling to occur. In fact, the establishment of transient adhesion points between colliding cells has been observed *in vitro* before their lamellipodia are retracted owing to CIL [38]. Moreover, it is clear that adhesion molecules do not only provide mechanical adhesion but they also work as ligand/receptor pairs playing an important role in cell signalling.

One of these molecule families is cadherins. Cadherins are a multigene family of cell surface glycoproteins that mediate Ca^2+^-dependent homophilic cell–cell adhesion by their extracellular domains. Moreover, they can activate intracellular signals involved in cell polarity and in cytoskeleton control such as RhoA and Ena/VASP [39]. Cadherins were among the first molecules that were directly implicated in CIL*.* E-cadherin has been shown to be required for CIL, and interestingly not for contact inhibition of proliferation (CIP), in migratory cells and in confluent epithelial cells [7,8,40,41]. Likewise, the XB/U-cadherin in *Xenopus* (similar to the mammalian P-cadherin) is required for the contact-dependent coordination in collective migration of head mesoderm [6]. Interestingly, a recent similar study in fish embryos has shown the requirement of E-cadherin for collective migration of mesoderm *in vivo* [42]. Although the authors mostly attribute its role to cell–cell adhesion, they also suggest a possible role for CIL in mesoderm migration. An appealing hypothesis would be that both adhesion and CIL converge at the level of E-cadherin to control collective migration. This is supported by the role of E-cadherin in CIL and the link between CIL and adhesion. Altogether, these data indicate that cadherins are essential for CIL. At the same time they highlight that different tissues or cell types could use different cadherins or even other adhesion molecules during CIL (Box 3).

### Molecular bases of CIL-dependent cell polarity

A key step in CIL is the inhibition of cell protrusions and the re-setting of the intracellular polarity. Cell protrusions are dynamic and complex structures that are formed largely by actin filaments and are regulated by intricate molecular networks (see Box 2, Ref. [43]). Thus, the inhibition of cell protrusions is not a passive mechanism but, instead, requires the activation of a complex regulatory machinery [44,45]. Members of the family of small Rho GTPases are essential in the control of both cell polarity and protrusions [46] (Box 2). RhoA is known to control myosin II-dependent contraction of the trailing end of a cell through the protein kinase ROCK [47,48]. Therefore, RhoA can be a negative regulator of protrusion formation and, thus, a good candidate for a protrusion inhibitory mechanism. The spatial and temporal control of RhoA activity is crucial for appropriate inhibition of cell protrusions. This control can be exerted by a variety of molecules, among which, we find cell adhesion molecules and members of the non-canonical Wnt or planar cell polarity (PCP) signalling pathway.

Recent evidence in neural crest cells shows that RhoA is involved in CIL via the PCP pathway [9]. Here it was shown that CIL members of the PCP signalling would be activated at cell–cell contacts, which in turn locally activate RhoA. Activated RhoA would then antagonise Rac1 and inhibit cell protrusions [9,34]. Interestingly, a similar mechanism has been described for the inhibition of cell protrusions by cell–cell contact during vasculogenesis [33]. Although this is an interesting mechanism, it does not fully account for how the presence of an adjacent cell is transduced into this intracellular mechanism. However, with the literature available it is possible to propose a molecular link between cadherins and RhoGTPases.

### Linking cadherins with Rho

Activation of different Rho GTPases appears to be cell type and cadherin-type dependent. It is well established that cadherin engagement leads to activation of Rac1 and Cdc42 and inhibition of RhoA at the cell contact region of many cells [49]. However, activation of RhoA as a result of cell–cell adhesion has been reported in keratinocytes [50] and in N-cadherin dependent cell–cell adhesion of C2C12 myoblasts [51]. It has also been shown that association of N-cadherin with p120 (a catenin that binds to the intracellular domain of cadherins) in cholesterol-rich microdomains leads to activation of RhoA during myogenesis [52]. Whether a similar mechanism occurs in migratory cells remains unknown. It is known that one of the functions of p120ctn is the regulation of Rho GTPases, which has led to suggest that the inhibitory activity of p120ctn on RhoA is dependent on the cytoplasm localisation of p120ctn. Expression of different cadherins sequestrates p120ctn to the membrane and blocks the inhibition of RhoA by p120ctn, suggesting that formation of cadherin-based cell–cell adhesion established during CIL sequesters p120ctn to the membrane and away from cytoplasmic pools therefore relieving the inhibition of RhoA activity (Refs [53–56]). In addition, it has been shown that inhibition of sprouting during vasculogenesis requires VE-cadherin, which in turn activates RhoA and inhibits Rac1 at cell junctions, in a process reminiscent of CIL [33,57].

It is probable that different cells use different molecules to interact with their neighbours, giving more versatility to CIL. This greater flexibility could explain why the same cell can exhibit CIL with one particular kind of cell but not with others. Other molecules such as Ephrins/Eph and Notch/Delta and PCP proteins are also possible mediators of CIL (Box 3).

## CIL in disease

The first crucial contribution of CIL to cancer research was the idea that cell locomotion is a normal activity of somatic cells that needs to be restricted for the cells to remain at the right place within an organism. This restriction comes from neighbouring cells, which when absent, will allow the migration of cells liberated from this repression. Malignancy, thus, is not necessarily the acquisition of motility by cancerous cells but the absence or lessening of the response to the inhibition of migration exerted by neighbours [3,4]. This intrinsic but inhibited tendency to migrate of at least some somatic cells has been proposed as important for normal physiological processes such as wound healing of epithelia. In addition to CIL the activity of growth factors also plays a role to stimulate re-epithelization and to produce significant changes in adhesion and cell morphology during wound healing [58,59].

**Figure 3:**
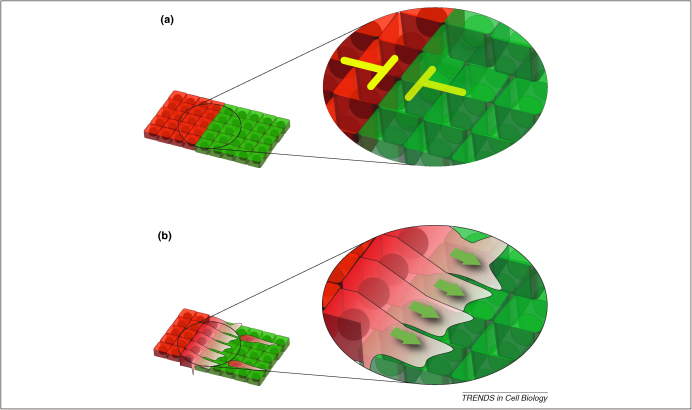
CIL in normal and cancer cells. **(a)** Two cell populations (indicated by two different colours) exhibit mutual CIL (yellow inhibitory arrows). This prevents the mixing of cells from these two populations. This kind of behaviour can be found in normal tissues. **(b)** Two cell populations are confronted and one of them (red cells) has lost CIL with the other (green cells). As a consequence the first group invades the second one. This invasive behaviour can be found in many cancer cells and has been proposed as the basis for metastasis.

To better understand the effect of CIL in normal and cancerous cells, it is necessary to keep in mind that there are two types of CIL. When two cells (or groups of cells) of the same type encounter each other they can exhibit CIL or not. If they do, it is said that they have **homotypic CIL** (see Glossary). This has been extensively documented in chick heart fibroblast [2], among other cell types. Similarly, two different cell types can encounter each other and also display CIL, which would then correspond to **heterotypic CIL** (see Glossary; Figure 3a). Chick heart fibroblasts, for example, exhibit this behaviour when confronted with normal mouse muscle fibroblasts [3].

By contrast, several sarcoma and melanoma cell lines have diminished or absent CIL: they will invade territories populated by other cells, such as normal fibroblasts (Figure 3b). This led to the conclusion that absence of CIL between tumour and normal cells was at the basis of invasive metastasis [3]. A common misunderstanding of these observations is to believe that malignant cells have lost CIL between themselves. It has been shown that malignant cells have lost heterotypic CIL when confronted with normal fibroblasts but they usually do not lose homotypic CIL (Ref. [4], P. Friedl, personal communication). This is similar to what happens to neural crest cells that have homotypic CIL (among neural crest cells) but can invade mesoderm and other tissues during their migration [9]. An appealing hypothesis would be that the invasive behaviour of tumours is facilitated by the absence of heterotypic CIL with normal cells, whereas homotypic CIL between cancer cells helps collective migration and/or dispersion of the tumour. Also, it should be noted that CIL in malignant cells is not always lost but sometimes diminished. For example, the invasion of S180 sarcoma cells is almost completely unobstructed by chick heart fibroblasts, whereas that of mouse melanomas or BAS56 sarcoma cells is partially blocked [3]. The implications of CIL in the invasive properties of tumours *in vivo* remain to be studied. A recent study showed that the activation of the cytoplasmic form of the human oncogene *MET* in mouse liver progenitor cells (MLP29) produced loss of contact inhibition [60]. Interestingly, when these cells are transplanted into the spleen of immunodeficient mice they become highly invasive carcinomas.

The usual misconception that cancer cells have lost contact inhibition only holds true for CIP (see Glossary) and not for CIL between cancer cells. It has been proposed that CIP and CIL are molecularly similar [61], but the evidence for this is scarce, and the simple observation that many tumours do not have CIP but retain CIL with other cancer cells, strongly suggests that these are two different processes. The molecular basis of CIL in cancer is a fascinating, but poorly developed, area of research that could have important practical implications. Understanding the molecular mechanism by which cancer cells have lost CIL with neighbouring cells could lead to new diagnostic and therapeutic tools.

## Concluding remarks and future directions

Collective migration is now a well-recognized mechanism of migration both in morphogenesis and in cancer progression [12,13,62]. Thus, the cellular and molecular understanding of the cell–cell interactions in this type of migration is crucial. CIL is a cellular interaction likely to be crucial for collective migration. However, a better description of its molecular basis *in vivo* is needed, as well as an understanding as to whether CIL is actually linked to collective cell migration (see Box 4 for outstanding questions about CIL).

At the same time development of fluorescent transgenic animals and improvements in microscopy techniques, such as confocal microscopy and time lapse imaging, will help to accelerate our molecular description of CIL *in vivo.* These techniques provide non-invasive imaging tools that allow us to investigate cell migration *in vivo* in genetically modifiable organisms where the role of different molecules can be tested*.*

These are early days in understanding the molecular basis of CIL. However, we have dissected it into two sequential mechanisms with different molecular players. They involve a cell adhesion molecule-dependent first step in which the colliding cells sense each other. This mechanism is followed by a repolarization of the cell. We provide evidence favouring a role for cadherins in the first process, followed by a RhoGTPases-dependent repolarization during the second. Also, we have proposed a link between these two processes. However, other molecules could also be mediating CIL and further investigation is needed to establish a detailed molecular explanation of CIL and to elucidate its cell-type specificity. This is a crucial step because it would allow us to investigate the problem of why most cancerous cell can reduce the heterotypic CIL while maintaining homotypic CIL among them.

The role of CIL in collective migration also raises an interesting problem of how large groups of cells can self-organize from local cell–cell interactions. Self-organization of migratory groups of cells have been described in several morphogenetic movements such as the lateral line [29] and the migration of tumour cohorts [12]. Thus, CIL together with other local interactions such as cell–cell adhesion could organise tissues at a much larger scale. In fact, recent mathematical models have successfully reproduced the behaviour of migratory sheets by only considering local interactions at the single cell level, especially CIL [63]. These cell–cell interactions can complement other mechanisms, such as taxis and physical forces, in promoting migration of groups and clusters of cells. The molecular characterization of CIL is just starting to be unravelled but the increasing evidence of its importance will promote a better understanding of this process in normal and pathological cell migration.

## Figures and Tables

**Figure I:**
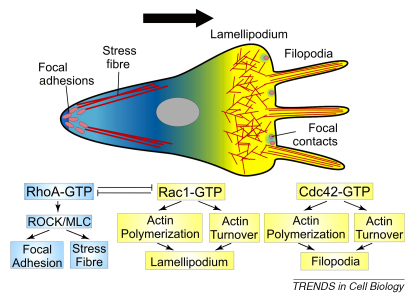
Rho GTPases and cell protrusion control.

## Box 1 The discovery of CIL

The concept of CIL gradually emerged from the work of Abercrombie and Heaysman starting in 1953. They wanted to study how the behaviour of a cell is influenced by other cells, i.e. their ‘*social behaviour*’.

Heaysman and Abercrombie observed what happened when two embryonic chicken heart explants were placed in close proximity. Collisions occur between fibroblasts migrating from opposing explants. At the time they started their experiments, they could not perform detailed microscopic observations. Instead, they did careful macroscopic measurements obtaining statistical parameters to describe cell behaviour. Nonetheless, they made at least two crucial observations. First, they observed that fibroblasts will lower their speed in proportion to the number of cells they encounter. Thus, contact with or the proximity to other cells will reduce cell motility [1]. Moreover, they observed that at the region where the two explants encountered each other, the fibroblasts would never clump on top of each other. Instead, they will halt their migration or disperse elsewhere [2]. They concluded that a cell would preferentially adhere to the substrate rather than to its neighbouring cell. They called this restriction CIL and proposed that the tendency of alignment between neighbouring cells and the monolayering of explants were outcomes of CIL [2].

Later on, with improved observation tools, they added important cellular details to the process such as the retraction of the cell protrusion after contact and that the ruffling activity of the membrane will be restarted elsewhere in the cell perimeter [64]. Thus, CIL will more often lead to the redirection of colliding fibroblasts than to stop their movement [4,38]. All these observations integrated a more complete definition of CIL: ‘*the phenomenon of a cell ceasing to continue moving in the same direction after contact with another cell*’ [3].

During the following years, different degrees of CIL were found in a large number of healthy and cancerous cell types (reviewed in Ref. [3]). Efforts were exerted to try to observe CIL *in vivo* with only limited success [10,11]. At the same time the implications of CIL in migration and morphogenesis were disputed [65–67]. By contrast, important advances in the field of cell migration were made with the identification of several molecular components that allowed the generation of models of cell polarization and chemotaxis [45,68–70]. These important new discoveries on directional cell migration were unmatched by a sufficiently detailed molecular understanding of CIL and the initial excitement on CIL faded away.

## Box 2 Rho GTPases and cell polarity

Directional cell migration is dependent on cell polarity which influences the formation of the leading and trailing cell edges. A typical polarized migrating cell exhibits cell protrusions, such as filopodia and lamellipodia, at the front and large focal adhesion complex at the back (Figure I). Directional cell migration is achieved by the polarized formation of cell protrusions at the front and the contraction of stress fibres at the trailing edge. The typical Rho GTPases – RhoA, Rac1 and Cdc42 – play a crucial role in controlling cell polarity. These three Rho GTPases regulate different aspects of cytoskeleton dynamics. Cdc42 has been shown to be involved in controlling the actin cytoskeleton present in protrusions known as filopodia [71]. Rac1 promotes the formation of lamellipodia – large, flattened and ruffling protrusions – by regulating actin polymerization [72]. The three Rho isoforms – RhoA, RhoB and RhoC – can induce stress fibre formation [73]. The general view is that in polarized cells Rac1 and Cdc42 are active at the front where they promote the formation of cell protrusions, whereas RhoA is active at the back where it controls cell contraction. In addition a clear mutual inhibition between Rac1 and RhoA has been established [74]. However, recent studies have shown that all three GTPases can be activated at the front of migrating cells, where RhoA has a role in the initial events of protrusion, whereas Rac1 and Cdc42 are involved in reinforcement and stabilization of newly expanded protrusions [75,76].

In addition to their role in actin dynamics, the Rho GTPases also control polarized adhesion to the substratum during directional migration. Small focal complex structures are localized in the lamellipodia of most migrating cells, and are important for the attachment of the extending lamellipodium to the extracellular matrix [68]. It has been shown that Rac is required for focal complex assembly [74,77]. Focal complexes can be disassembled as the cell lamella moves over them or can mature into focal adhesions induced by RhoA [74].

For many cells the final step of the cell migration cycle is the retraction of the back to move forward. This cell body contraction is dependent on actomyosin contractility and can be regulated by RhoA via ROCKs (also known as Rho-kinases) to affect myosin light chain (MLC) phosphorylation, both by inhibiting MLC phosphatase and by phosphorylating MLC [78].

## Box 3 Possible surface molecules that control CIL

Candidates for cell–cell recognition required in CIL should be transmembrane molecules involved in contact-mediated cell signalling. They also should control cell protrusion formation/retraction, probably via the regulation of the cytoskeleton dynamics. Cadherins accomplish these criteria (see main text) but other molecules could also have a potential role in CIL.

Another group of CIL candidate molecules are the **atypical cadherins** Dachsous (Ds), Fat and Flamingo (Fmi), which are involved in planar cell polarity (PCP) or non-canonical Wnt signalling. Because Ds, Fat and Fmi play crucial roles in the localization of the typical PCP receptor Frizzled (Fz), [39] it is possible that cell collision during CIL engages these atypical cadherins, which in turn activate Fz and Dsh in the region of cell contact. Interestingly, it has been shown that Fmi plays a crucial role in cell movements and migration during zebrafish gastrulation [79]. However, the role of these atypical cadherins in CIL needs to be directly tested.

The ***Notch-Delta*** signalling pathway is usually known by its transcriptional regulation that occurs after Delta, a transmembrane protein binds its receptor Notch in an adjacent cell. This leads to the cleavage of Notch whose intracellular domain is translocated to the cell nucleus [80,81]. However, there are recent data in *Drosophila* for non-transcriptional branches of the Notch signalling [82,83]. This non-transcriptional function is based on the interaction of Notch with the intracellular tyrosine kinase Abl and its cofactors, crucial for the motility and guidance of motor axons (Refs [84–86]). Interestingly, one of the molecules that interacts with Notch and Abl is Trio, a guanine nucleotide exchange factor (GEF) protein that can activate RhoA [87,88].

***Ephrins*** comprise a family of transmembrane proteins that bind to their cognate receptor tyrosine kinase Eph. Both ephrins and Eph receptors can transduce signals and they are crucial regulators of cell–cell communication in processes like cell adhesion and repulsion [89]. The repulsive activity of Eph/ephrin signals has been well characterized in axonal guidance. It is possible that this repulsive activity could be similar in molecular terms to the collapse of protrusions in CIL.

There are two classes, A and B, of Ephrins, which usually interact with EphA and EphB receptors, respectively. They can activate Rho GTPases, such as RhoA (preferentially via ephrinA/EphA binding) and Rac1 and Cdc42 (preferentially via ephrinB/EphB binding, Ref. [90]). Nonetheless, ephrinB/EphB can also activate RhoA via Dsh/PCP signalling [91–93].

***Nectins*** have various roles in cell polarization, differentiation, movement, proliferation and survival and they have been directly implicated in contact inhibition [61]. They are known to inhibit RhoA, in an Src dependent manner, by a ternary complex formed by Necl5, integrin αvβ3 and PDGFR. It has been proposed that cell–cell contact allows the interaction between Nectin3 and Nectin-like 5 (Necl5) which leads to endocytosis of Necl5, so releasing RhoA inhibition [61]. However, it has not been properly clarified if this mechanism leads to the inhibition of proliferation or to proper CIL.

## Box 4 Outstanding questions

• What are the signals that mediate cell–cell recognition during CIL?
• How are homotypic and heterotypic CIL controlled?
• How is the change in cell polarity controlled during CIL?
• How is CIL related with other cellular processes such as axon guidance/repulsion?
• Is the inhibition of cell protrusions observed in collective cell migration dependent on CIL?
• How does CIL modulate the contribution of other mechanisms in migration, such as adhesion, taxis and invasion?
• How is CIL involved in cancer metastasis?

Finally, a better understanding of CIL will contribute to our understanding of wound healing and contribute towards strategies to control cancer cell invasion.

## Glossary

Contact inhibition of locomotion (CIL): the phenomenon of a cell ceasing to continue moving in the same direction after contact with another cell.
Contact inhibition of proliferation (CIP): the phenomenon of a cell ceasing to proliferate after contact with other cells.
Heterotypic contact inhibition of locomotion: CIL present between cells of different types. It is usually lost in cancer cells when confronted with normal cells.
Homotypic contact inhibition of locomotion: CIL present between cells of the same type. It is exhibited by many normal and cancer cells.
Neural crest: A stem cell-like, migratory population of embryonic cells that forms laterally to the prospective central nervous system. It is essential for vertebrate development because of the wide range of derivatives to which it gives rise.
Endodermal cells: cells composing the inner-most embryonic germinal layer, that is the endoderm. These cells will differentiate mostly into the respiratory and digestive tracts of the adult body.
Myeloid cells: Blood cell precursors. In vertebrates there are two waves of haematopoiesis (the generation of blood cells). The first one, and the one relevant for the movements described in this article, is an embryonic (primitive) haematopoiesis in which blood cell precursors disperse from specific regions of the embryo to populate the entire body.
Cajal-Retzius cells: Transient neuronal population crucial for the development of the brain cortex.
